# Editorial: New advances in functional aquafeeds

**DOI:** 10.3389/fvets.2023.1187586

**Published:** 2023-04-03

**Authors:** Ghasem Rashidian, Ndakalimwe N. Gabriel, Ramesh Dharmaraj

**Affiliations:** ^1^Faculty of Fisheries and Protection of Waters, South Bohemian Research Center of Aquaculture and Biodiversity of Hydrocenoses, Institute of Aquaculture and Protection of Waters, University of South Bohemia in České Budějovice, České Budějovice, Czechia; ^2^Department of Fisheries and Ocean Sciences, Faculty of Agriculture, Engineering, and Natural Resources, University of Namibia, Windhoek, Namibia; ^3^School of Aquaculture and Aquatic Sciences, College of Agriculture, Communities, and the Environment, Kentucky State University, Frankfort, KY, United States; ^4^Central Institute of Freshwater Aquaculture (ICAR), Bhubaneswar, India

**Keywords:** functional feed, feed additives, phytochemicals, immunostimulants, growth promotors, pre and probiotics

The aquaculture industry is increasingly growing every year underlining the demand for more effective aquafeeds. Feed additives have become an integrated part of manufacturing aquafeeds which are used for several purposes such as promoting growth, enhancing immunity, and improving resistance against diseases. Feed additives, particularly those of natural origin, are chosen since they are more sustainable than pharmaceutical synthetic drugs used in aquaculture for the same reason, and it is speculated that a combination of feed additives can elicit stronger responses. This is important in the part where different sets of additives can be used to stimulate different aspects of fish physiology. This Research Topic focuses on how feed additives can help provide farmed fish with functional feed and report their mechanisms of action.

The primary functional feed additives presented in this Research Topic are phyto-additives (of plant origin). Mbokane and Moyo contributed a review on the current achievements in the use of plant-origin feed additives particularly in freshwater aquaculture in Southern Africa and highlighted their availability and efficacy in *Oreochromis mossambicus* and *Clarias gariepinus*. This review shows that plants can be used as feed additives to improve overall fish production through improved growth, disease resistance, and ability to control reproduction. However, their studies indicated that the use of feed additives in Southern Africa is still limited to experiments; there is a lack of application at the farm level. The authors strongly recommended plants as feed additives, especially in the small-scale sector where production is hampered by the poor quality of feeds, precocious breeding in tilapia fish, poor condition of breeders, and disease outbreaks.

Another review by Kari et al. outlined the effects of phytobiotics against *Aeromonas hydrophila*, the causative agent of motile aeromonad septicemia (MAS) in fish. This scientific contribution provides valuable information regarding the plant-based active substances capable of stimulating fish growth, immunity, and resistance against *A. hydrophila* which can help aquaculture reduce its traditional reliance on antibiotics to fight diseases. The practical and reliable published results can be exploited by local farmers in small-scale production sectors to approve their efficacy.

A study by Fath El-Bab et al. tested the synergistic effects of β-glucan and/or Bacillus coagulants in Nile tilapia (*O. niloticus*) whereby the fish that received both additives exhibited greater positive responses in terms of growth performance, immune, and antioxidant parameters. Furthermore, mRNA expression levels of heat shock protein-70 (HSP70), growth hormone (GH), interleukin-1 beta (IL-Iβ), and interleukin-8 (IL-8) genes in the liver tissue of Nile tilapia were strongly affected when both additives were incorporated. It is worth mentioning that the intestine of fish receiving both additives had the longest villi length, and overall fish intestine was found healthy with signs of inflammation.

The last study was by Dawood and Shi investigated the effect of dietary β-mannanase supplementation on growth performance, digestibility, and gene expression levels of *Cyprinus carpio* (Linnaeus) fingerlings fed a plant protein-rich diet. This study recommended β-mannanase supplementation (500 U/kg) for obtaining better carp production especially when low-cost plant-protein rich diets are used.

We believe that the research outputs in this Research Topic will give readers insight into new breakthroughs in functional feed additives used in aquafeeds to improve aquaculture production in a sustainable approach.

## Author contributions

GR: manuscript drafting and final write-up. NG and RD: manuscript review and final write-up. All authors contributed to the article and approved the submitted version.

